# Prevalence of myositis specific and associated antibodies in a cohort of patients affected by idiopathic NSIP and no hint of inflammatory myopathies

**DOI:** 10.1007/s12026-023-09387-z

**Published:** 2023-05-03

**Authors:** Edoardo Conticini, Miriana d’Alessandro, Paolo Cameli, Laura Bergantini, Elena Pordon, Lucia Cassai, Luca Cantarini, Elena Bargagli, Bruno Frediani, Brunetta Porcelli

**Affiliations:** 1 Department of Medicine, Surgery & Neurosciences, Rheumatology Unit, University of Siena, 53100 Siena, Italy; 2 Respiratory Diseases Unit, Department of Medical and Surgical Sciences & Neurosciences, University of Siena, Viale Bracci 1, 53100 Siena, Italy; 3 UOC Laboratorio Patologia Clinica, Policlinico S. Maria Alle Scotte, AOU Senese, Siena, Italy; 4 Dipartimento Biotecnologie Mediche, Università Degli Studi Di Siena, Siena, Italy

**Keywords:** Non-specific interstitial pneumonia, Autoantibodies, Idiopathic inflammatory myopathies

## Abstract

The presence of interstitial lung disease (ILD) is a common and fearsome feature of idiopathic inflammatory myopathies (IIM). Such patients show radiological pattern of non-specific interstitial pneumonia (NSIP). The present study aimed to assess the prevalence of myositis-specific and myositis-associated antibodies (MSA and MAA) in a cohort of patients with a previous diagnosis of NSIP and no sign or symptom of IIM. Secondly, it will be assessed whether patients displaying MSA and/or MAA positivity have a worse or a better outcome than idiopathic NSIP. All patients affected by idiopathic NSIP were enrolled. MSA and MAA were detected using EUROLINE Autoimmune Inflammatory Myopathies 20 Ag (Euroimmun Lubeck, Germany), line immunoassay. A total of 16 patients (mean age 72 ± 6.1 years old) were enrolled. Six out of 16 patients (37.5%) had significant MSA and/or MAA positivity: one displayed positivity of anti-PL-7 (+ +), one of anti-Zo (+ +), anti-TIF1γ (+ + +) and anti-Pm-Scl 75 (+ + +), one of anti-Ro52 (+ +), one of anti-Mi2β (+ + +), one of anti-Pm-Scl 75 (+ + +) and the latter of both anti-EJ (+ + +) and anti-Ro52 (+ + +).

Two out of 7 seropositive patients showed a significant impairment of FVC (relative risk 4.8, 95% CI 0.78–29.5; *p* = 0.0350). Accordingly, among the 5 patients that started antifibrotic treatment during the observation time, 4 were seronegative. Our findings highlighted a potential autoimmune or inflammatory in idiopathic NSIP patients and also in those without significant rheumatological symptoms. A more accurate diagnostic assessment may ameliorate diagnostic accuracy as well as may provide new therapeutic strategy (antifibrotic + immunosuppressive). A cautious assessment of NSIP patients with a progressive and non-responsive to glucocorticoids disease course should therefore include an autoimmunity panel comprising MSA and MAA.

## Introduction

Pulmonary involvement, occurring through interstitial lung disease (ILD), is one of the most common and fearsome features of anti-synthetase syndrome (ASS), a systemic disease characterized by the positivity of anti-aminoacyl-transfer-RNA synthetase antibodies (ARS), systemic symptoms and a typical involvement of lung, muscle, skin and joints. ILD, due to the progressive clinical course, leading to respiratory failure and death, represents a negative prognostic factor [1] and requires a prompt diagnosis and treatment, which can stabilize pulmonary function. On this topic, the concept of progressive pulmonary fibrosis (PPF) has been recently proposed to describe all those clinical entities, including connective tissue diseases with ILD (CTD-ILD), that may develop, in a relevant percentage of patients, a progressive and inexorable decline of respiratory function leading to chronic respiratory failure and death, mimicking the behavior of idiopathic pulmonary fibrosis (IPF) [2]. Among CTD, ASS is significantly more likely to exhibit ILD compared to dermatomyositis (DM) and polymyositis (PM), especially for patients who are positive for anti-Jo-1 [2]. Conversely, certain other myositis-specific antibodies (MSA), such as TIF1γ and Mi2, are negatively associated with the presence of ILD [3]. At the same time, certain MSA, such as SAE, are associated to a more severe cutaneous disease, while some others, such as TIF1γ, to the presence of malignancy, therefore displaying a worse prognosis and a poor response to treatment. In this regard, the diagnostic work-up of a patient with suspected IIM cannot be separated by a meticulous assessment of autoimmune profile.

Most ASS, in which ILD can be detected in up to 80% of patients [4, 5], have a non-specific interstitial pneumonia (NSIP) pattern; usual interstitial pneumonia (UIP) is more common in anti-PL7 + patients, whose outcome is generally worse, while organizing pneumonia (OP) accounts for up to 78% among EJ + ones [6], displaying nevertheless a good response to treatment. Indeed, UIP pattern, although less common, seems to account for a worse prognosis [7], as well as the presence of fever and a lower peripheral CD3 + /CD4 + percentages [8].

Nevertheless, differently from IPF, ASS-ILD, including UIP, has a better prognosis, displaying a higher survival rate [9], despite undistinguishable imaging and histological features.

It has been demonstrated that patients with NSIP pattern showed a more relevant respiratory functional impairment in terms of forced vital capacity (FVC) and diffusion lung capacity for carbon monoxide (DLCO) than UIP ones [3]. Nevertheless, if rapidly treated, prognosis of ASS-NSIP is usually favorable, displaying a good response to treatment [10].

In this regard, a tempestive diagnosis of ASS, particularly in case of concomitant ILD, has a paramount role in increasing survival and remission rate in these patients. Diagnostic delay is still considerable (29, 11–63 months) [11] both for a poor awareness of this protean condition and the low availability of diagnostic tools such as antibodies.

International guidelines for diagnosis of IPF and PPF underline the importance to reach a specific diagnosis for the correct management of ILD patient (2): therefore, considering the significant heterogeneity of rheumatological symptoms in patients with CTD-ILD, both in terms of specificity and time of onset, the potential application of new biomarkers that could widen our diagnostic performance is absolutely intriguing.

Thus, the aim of this study was to assess the prevalence of MSA and myositis-associated antibodies (MAA) in a cohort of patients with a previous diagnosis of NSIP and no sign or symptom of IIM.

Secondary endpoints were to compare clinical, respiratory functional and imaging features between seronegative and seropositive NSIP patients and to evaluate and whether patients displaying MSA and/or MAA positivity have a worse or a better outcome than idiopathic NSIP.

## Materials and methods

### Patients

We retrospectively collected and analyzed serum samples from patients with idiopathic NSIP followed at Siena Regional Referral Centre for ILD whose diagnosis was made from January 2017 to January 2020.

Inclusion criteria were: a definite radiological evidence of NSIP at chest high resolution computed tomography (HRCT), performed by a radiologist experienced in this field; a diagnosis of idiopathic NSIP, performed through a multidisciplinary discussion, including at least a pulmonologist, a radiologist and a rheumatologist, all with a specific expertise in ILD; the availability of a minimum core set measures (age, sex, date and type of symptoms, date of diagnosis, complete pneumological evaluation, ESR, CRP, CK, transaminase, LFTs); the availability of a serum sampling (performed within 6 months the diagnosis of NSIP) for autoimmunity assay; the lack of any sign or symptom suggestive for any rheumatic or autoimmune disease, including serological testing performed during diagnostic pathway.

Exclusion criteria were: a diagnosis of interstitial pneumonia with autoimmune features (IPAF), a UIP pattern at chest HRCT, the lack of any of the above mentioned data, the unavailability of serum collected at the time of first diagnosis, a previous treatment with any antifibrotic and immunosuppressive agent, except glucocorticoids, a diagnosis of neoplasia within 5 years from serum collection and the presence of any of the following signs or symptoms, at the time of the assessment or before: unexplained fever and/or weight loss, arthritis, dysphagia, dysphonia, Raynaud’s phenomenon, mechanic’s hands, hiker’s feet, sclerodactyly, malar rash, Gottron’s papules, heliotrope rash, V-sign, shawl sign, calcinosis, positivity of rheumatoid factor (RF) and/or anti-citrullinated peptide (CCP) antibodies, proximal muscle weakness, CK and/or myoglobin and/or aldolase increase > 2 the upper normal value repeated in two consecutive assessment.

All patients underwent clinical and respiratory functional follow-up according to our Centre protocol for fibrotic ILDs: survival outcomes and all pulmonary functional tests parameters performed at our Centre during the observation time were retrospectively collected for statistical analysis. The respiratory functional assessment was performed within 1.7 ± 3.1 months of the serum sampling, while BAL cellular analysis was available only for 3 patients (all females) and was performed for diagnostic purposes.

## Chest HRCT features

Chest HRCT was performed in all patients and interpreted by experienced thoracic radiologists.

In case of HRCT performed elsewhere, images were acquired and collectively discussed, prior agreement in terms of quality of the scan.

## Autoimmunity assay

MSA and MAA were detected using EUROLINE Autoimmune Inflammatory Myopathies 20 Ag (Euroimmun Lubeck, Germany), line immunoassay which provides qualitative determination of autoantibodies of the immunoglobulin class IgG to 20 different antigens: Mi-2α, Mi-2β, TIF1γ, MDA5, NXP2, SAE1, Ku, PM-Scl100, PM-Scl75, Jo-1, SRP, PL-7, PL-12, EJ, OJ, Ro-52, cN-1A, Ha, Ks and ZO. The test kit contains test strips coated with parallel lines of highly purified antigens. In the first reaction step, the immunoblot strips are incubated with diluted patient samples (1:101 in sample buffer) by automated incubation (EUROBlotOne). In the case of positive samples, the specific IgG antibodies (also IgA and IgM) will bind to the corresponding antigenic site. To detect the bound antibodies, a second incubation is carried out using an enzyme-labeled anti-human IgG (enzyme conjugate) catalysing a color reaction. The imaging and evaluation are possible directly from the incubation trays. EUROIMMUN recommends interpreting results based on the signal intensity: no signal (0): negative; very weak band ( +): borderline; medium to strong band (+ , + +): positive; very strong band with an intensity comparable to the control band: strong positive.

The line blot was analyzed by a single biologist with 30 years of experience in the field of autoimmunity.

## Diagnostic criteria

After the autoimmunity assessment, all patients were re-evaluated in order to assess whether they could satisfy any of the following criteria: EULAR/ACR for IIM [12] and Connors [13], Solomon [14] and Lega [15] for ASS.

## Statistical analysis

Patients’ characteristics were reported using median and interquartile range (IQR) for the quantitative variables, and absolute/relative frequency values for qualitative variables. The clinical outcomes, safety and treatments differences were examined by Chi-square, Kruskal–Wallis, Fisher or Mann–Whitney *U* test, when appropriate. Kaplan–Meier and Log-rank test were used to estimate survival and evaluate differences between subgroups. A *p* value < 0.05 was considered significant. Analysis was carried out using SPSS/GraphPad/STATA.

## Ethics

The study was carried out in accordance with the Declaration of Helsinki and its amendments and was approved by the local ethical committee (Comitato Etico Area Vasta Sud Est, Tuscany code number 180712 and Markerlung, code number 17431).

## Results

### Study population and respiratory functional assessment

A total of 16 patients (8 males, 8 females, mean age at the time of sample collection 72 ± 6.1 years old, mean duration of disease 46.6 ± 40.5 months) were included in our study.

At the time of the assessment, patients showed a mild to moderate restrictive impairment of lung volumes, paired with a moderate reduction of DLCO, on average. LFTs findings are summarized in Table 1.

**Table 1:** Demographic, clinical and immunological features of study population, stratified according to serological positivity

Parameter	Total population	Seropositive patients	Seronegative patients	*p* value
*N*°	16	7	9	
Female gender (%)	8 (50)	5 (71.4)	3 (33.3)	0.3147
Age (years)	72 ± 6.1	72 ± 6.6	72.2 ± 5.5	0.9096
*Therapy*				
Oral steroids (%)	16 (100)	7 (100)	9 (100)	1.0000
Mycophenolate (%)	4 (25)	1 (14.2)	3 (33.3)	0.5846
Azathioprine (%)	2 (12.5)	1 (14.2)	1 (11.1)	1.0000
Nintedanib (%)	5 (31.2)	1 (14.2)	4 (44.4)	0.3077
*Serological testing*				
CRP (mg/dl)	0.73 ± 1.07	0.96 ± 1.2	0.21 ± 1	0.2573
AST (UI/l)	22.5 ± 9.1	24.1 ± 9.5	16.6 ± 4.4	0.2129
ALT (UI/l)	21.6 ± 14.9	24.8 ± 16.7	12.8 ± 2.7	0.0534
LDH (UI/l)	195 ± 4.2	187.4 ± 6.4	197.1 ± 3.5	0.5986
CPK (UI/l)	44.5 ± 0.7	46.5 ± 3.4	42.1 ± 1.5	0.4968
Myoglobin (UI/l)	44.5 ± 2.1	47.1 ± 3.6	43.6 ± 3.6	0.8569
Aldolase (UI/l)	4.5 ± 1.9	4.4 ± 1.1	4.5 ± 1.3	0.8796
ANA titre				0.1296
- Negative (< 1:160)	11 (68.7)	3 (42.8)	8 (88.8)	
- 1:160–1:320	4 (25)	3 (42.8)	1 (11.1)	
- > 1:320	1 (6.2)	1 (14.2)	0 (0)	
*Lung function tests*				
*FVC ml*	2308.1 ± 962	2224.2 ± 1079	2373.3 ± 922.5	0.3510
FVC (%)	79 ± 18.7	83.1 ± 13.6	75.9 ± 22.2	0.7340
FEV1 ml	1865 ± 668.6	1774.2 ± 644.3	1935.5 ± 703.2	0.6806
FEV1 (%)	81.1 ± 16.9	84.2 ± 12.5	78.5 ± 20.3	0.6504
FEV1/FVC	82.6 ± 8.6	82.1 ± 10.7	83.1 ± 7.2	0.8371
DLCO (%)	59.1 ± 30.9	62 ± 10.6	57.1 ± 39.3	0.2977
KCO (%)	86.7 ± 34.6	94.1 ± 17.9	79.6 ± 42.6	0.5035
*Outcomes*				
Δ FVC ml	− 200.6 ± 280.9	− 61.4 ± 165.4	− 322.5 ± 313	0.1520
Δ FVC %	− 8.1 ± 11.5	− 3.3 ± 8.9	− 12.3 ± 12.4	0.1236
Δ DLCO ml/min/mmHg	− 2.9 ± 12.5	1.3 ± 2.4	− 6.4 ± 16.6	0.3602
Δ DLCO %	− 6.4 ± 8.5	− 4.7 ± 6.7	− 7.1 ± 9.4	0.5255
Death (%)	4 (25)	0 (0)	4 (44.4)	0.2335

*Abbreviation CRP* C-reactive protein, *AST* aspartate transaminase, *ALT* alanine transaminase, *LDH* lactate dehydrogenase, *CPK* creatine phosphokinase, *DLCO* diffusing capacity for carbon monoxide, *F* female, *FEV1* first second of forced expiration, *FVC* forced vital capacity, *KCO* carbon monoxide transfer coefficient, *M* male, *MAA* myositis-associated antibodies, *MSA* myositis-specific antibodies

No patient had evidence of myositis, arthritis, dysphagia or dysphonia nor gastrointestinal, cardiac or cutaneous involvement. None of them reported Raynaud’s phenomenon, while 3 of them complained from mild arthralgias and one from an episode of fever.

During the follow-up, the patients were treated with oral steroids mainly prednisone at the mean daily dosage of 12.5 mg, immunosuppressants like oral mycophenolate mofetil and, in patients with progressive interstitial lung involvement, nintedanib, at the daily routinary dosage of 150 mg twice a day.

Mean time of observation after serum sampling was 810.6 ± 597.9 days: four out of 16 patients (25%) died during follow-up due to chronic respiratory failure secondary to the progression of ILD.

## Laboratory values

Routine blood exams displayed slightly elevated values of CRP (0.73 ± 1.07 mg/dl) and normal transaminase (AST 22.5 ± 9.07 UI/l, ALT 21.06 ± 14.91 UI/l), LDH (195 ± 4.24 UI/l), CPK (44.5 ± 0.7 UI/l), myoglobin (44.5 ± 2.12 UI/l) and aldolase (4.55 ± 1.9 UI/l)** (**Table 1**)**.

Six out of 16 patients (37.5%) had significant MSA and/or MAA positivity: one displayed positivity of anti-PL-7 (+ +), one of anti-Zo (+ +), anti-TIF1γ (+ + +) and anti-Pm-Scl 75 (+ + +), one of anti-Ro52 (+ +), one of anti-Mi2β (+ + +), one of anti-Pm-Scl 75 (+ + +) and the latter of both anti-EJ (+ + +) and anti-Ro52 (+ + +).

## Correlation with clinical findings

No correlation was found between non-respiratory clinical symptoms and autoimmunity findings: in particular, 2 out of 3 patients suffering from arthralgias were seronegative.

We did not observe any significant differences regarding age, gender prevalence, time from diagnosis to serum sampling, respiratory functional parameters and serological markers (CPR, GOT and GPT) between seropositive and seronegative patients.

**Fig. 1:**
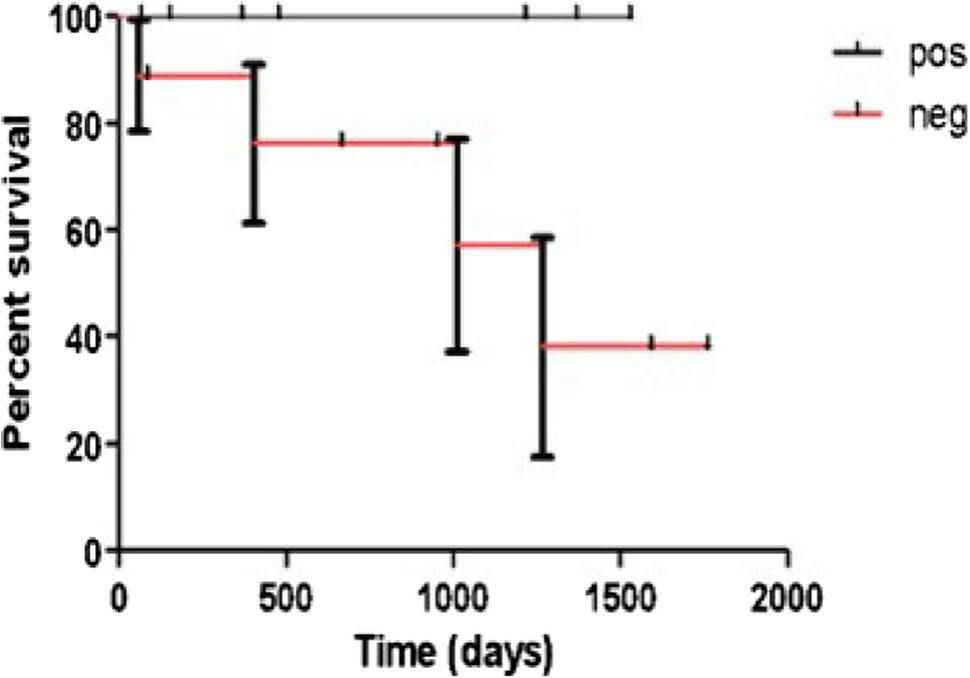
Survival comparison between seropositive and seronegative patients assessed through Kaplan–Meier curves (log rank test chi square 3.358; *p* = 0.0328)

Conversely, a robust correlation was found between serological profile and final outcome: all 4 patients who died or underwent lung transplant were seronegative, and such an unfavorable outcome was assessed in 4/9 (44.4%) of seronegative subjects (Fig. 1).

Follow-up PFTs, performed at least after 1 year from serum sampling, were available in 15 patients (7 serum-positive, 94% of the total population). Serum-negative patients showed a numerically more relevant decrease of FVC and DLCO during the follow-up than serum-negative (Table 1); according to the definition of PPF in non-IPF ILD patients, we observed that almost all seronegative patients fulfilled respiratory functional criteria for PPF (7 patients, 87.5%), while only 2/7 (28.5%) seropositive patients showed a significant impairment of FVC (relative risk 4.8, 95% CI 0.78–29.5; *p* = 0.0350). Accordingly, among the 5 patients that started antifibrotic treatment during the observation time, 4 were seronegative.

## Diagnostic criteria

Among the patients who displayed any MSA and or MAA positivity, 3 fulfilled Connors [12] and Lega [14] criteria for ASS, while none satisfied Solomon [13] and EULAR/ACR [11] ones.

## Discussion

In our study, we evidenced a high prevalence (37.5%) of MSA and/or MAA positivity in patients affected by idiopathic NSIP with no sign or symptom of any rheumatic disorder. In particular, 3 of them were ARS positive and formally fulfilled criteria for ASS, despite the lack of any cutaneous, muscular and systemic feature of IIM.

The increasing availability of novel biomarkers in the rheumatology setting has led to a more profound awareness of several autoimmune conditions. In particular, MSAs, although restricted to few, specialized, centers, allow both precocious diagnosis and risk stratification of IIM.

The knowledge of the different features and prognosis associated to each MSA should therefore address the clinician to a tailored and timely assessment of disease extent, followed by a prompt immunosuppressive treatment.

The rarity of these conditions makes it difficult for us to determine whether different forms of ASS may be caused by various ARS or whether all patients eventually suffer from the classical triad of ILD (arthritis, myositis and ILD): most published papers display highly contradictory results [16] in terms of incidence and timing of onset of the different clinical features. Nevertheless, ILD seems to occur in all patients affected by ASS, irrespective of autoimmune profile [17].

Moreover, the recent individuation of three novel autoantibodies, i.e. anti-Zo, Ks and Ha, has contributed to shed a new light in the comprehension of ASS. ILD seems to be one of the most common feature in anti-Ks and anti-Ha positive patients [18, 19], in whom lung involvement may be the sole manifestation of ASS and present with NSIP pattern and long-term stabilization of lung functionality [20]. Conversely, in case of anti-Zo, ILD, arthritis and myositis are reported in similar percentage [21, 22], but data are restricted to a dozen of patients [21–24].

Specifically focusing on these new antibodies (anti-Zo, Ks and Ha, plus anti-cN1A), a large study on 1194 ILD patients and 116 healthy controls evidenced a relatively high prevalence of such MSA, ranging from 0.9 to 2% [19]. In particular, an unclassified pneumonia accounted for up to 35% of patients positive for anti-Zo, Ks and Ha, while NSIP was diagnosed in 5.9% of patients positive for anti-Zo and 13.4% for anti-Ks.

In a previous study [25], conducted in a large Japanese cohort, ARS were detected in 13 out of 198 (6.6%) idiopathic interstitial pneumonia (IIP); nevertheless, these patients, although not fulfilling any criteria for CTD or vasculitis, suffered from systemic or organ-specific sign and symptoms potentially addressing through a suspect of myositis: indeed, a statistically significant difference was assessed in term of cutaneous symptoms (e.g. Gottron’s sign and heliotrope rash) between ARS positive and negative patients and two of them also displayed positivity for anti-CCP.

Another study [26], including all MSAs, and not only ARS, evidenced a prevalence of 17.6% in a cohort of IIP. Nevertheless, not only these patients had radiological features of IPAF, but 75% of the MSA positive ones had at least a hint of an underlying autoimmune condition.

Differently from the abovementioned papers, our study included fully asymptomatic patients from a rheumatological point of view, in whom a diagnosis of any CTD or IIM could not be reasonably suspected. At the same time, none of them received a confident or provisional diagnosis of IPAF at multidisciplinary discussion nor was positive for any other autoantibody, such as RF and anti-CCP. Finally, we decided to exclude patients with a radiological UIP pattern, thus specifically focusing on NSIP, which represents the most common CT pattern in CTD-ILD and whose definite diagnostic assessment is often challenging.

Aside from ARS, whose high prevalence was assessed in other studies [25, 26], two patients carried a positivity for MSA (Mi2 and TIF1γ) who are not classically associated with the occurrence of ILD [27]. Moreover, one patient displayed high-titer positivity for anti-Zo, TIF1γ and PmScl75: to the best of our knowledge, this is the first report of a triple positivity of these three antibodies.

Anyway, it is of interest that none of the patients displaying MSA/MAA positivity had a fatal outcome: none of them died or eventually underwent lung transplant, and even lung functionality showed a decline that, however, was almost significantly less pronounced in respect with seronegative patients. Even if observed in a very small cohort of patients, these findings are surely intriguing and further underline the clinical significance to discriminate between PPF with or without a specific cause, also for a more accurate prognostic estimation and therapeutic approach.

Moreover, despite the large observational period, it is noteworthy that none of the patients eventually diagnosed with ASS developed an extra-pulmonary involvement.

Overall, the present study further underscores the crucial importance of an accurate and high-experienced diagnostic approach on ILD patients, especially those with a radiological NSIP pattern. Despite the small sample size, the implementation of an updated serological testing led to relevant modification in terms of diagnostic and, consequently, therapeutic assessment, in a relevant percentage of patients with no signs or symptoms suggestive for CTD. Therefore, our data supports a working diagnosis approach for “idiopathic NSIP” and may also suggest to repeat an extended serological testing also in asymptomatic patients during the follow-up.

Our study has two main limitations: first, the relatively low number of patients, secondly the retrospective design, which does not allow to draw any firm conclusion in terms of prognosis and therapeutic options. Indeed, even though some patients have been treated with immunosuppressive agents throughout the follow-up, this pharmacological approach was not targeted to an underlying CTD, since it was meant as a adjunctive treatment for NSIP, as suggested prior the approval of nintedanib for PPF. Thus, we cannot assess whether a precocious and tailored immunosuppressive treatment would have led to a better outcome [28, 29]. Moreover, the strict exclusion criteria, which did not allow to include several patients from our cohort, strengthen our findings, but unavoidably excluded radiological pattern other than NSIP.

Finally, we are not allowed to firmly state whether MSA/MAA positivity is an epiphenomenon, in patients with NSIP, or whether the association of ILD and MSA, without any other sign or symptom, represents a definite subset of IIM, with a different severity, prognosis and response to immunosuppressive and antifibrotic agents.

In conclusion, our study, which is the first one employing a recently licensed 20 Ag blot for myositis in a cohort of NSIP patients with no sign and symptoms for IIM, evidenced a relevant prevalence of ARS, followed by the occurrence of antibodies not classically associated with the occurrence of ILD.

Our findings should lead to a more profound awareness of IIM in general and ASS in particular and should make reconsider the definition itself of “idiopathic NSIP”, for which an underlying potential autoimmune or inflammatory may be associated in a higher percentage than previously expected, also in patients without significant rheumatological symptoms. This assumption is surely intriguing and clinically relevant, since antifibrotic treatment is currently suggested only for those non-IPF patients showing a progressive course despite the therapy of underlying disease: therefore, a more accurate diagnostic assessment may not only ameliorate our diagnostic accuracy but may also provide new therapeutic opportunities in terms of combination therapy (antifibrotic + immune.suppressive), as recently described. A cautious assessment of patient with NSIP, in particular the ones with a progressive, non-responsive to glucocorticoids, but non-fatal, disease course, should therefore include an autoimmunity panel comprising MSA and MAA.
